# Association between antibiotic consumption and colon and rectal cancer development in older individuals: A territory‐wide study

**DOI:** 10.1002/cam4.4759

**Published:** 2022-04-29

**Authors:** Ka Shing Cheung, Esther W. Chan, Anthony Tam, Irene O. L. Wong, Wai Kay Seto, Ivan F. N. Hung, Ian C. K. Wong, Wai K. Leung

**Affiliations:** ^1^ Department of Medicine Queen Mary Hospital, The University of Hong Kong Hong Kong City Hong Kong; ^2^ Department of Medicine The University of Hong Kong&Shenzhen Hospital Shenzhen China; ^3^ Centre for Safe Medication Practice and Research, Department of Pharmacology and Pharmacy The University of Hong Kong Hong Kong City Hong Kong; ^4^ School of Public Health The University of Hong Kong Hong Kong City Hong Kong; ^5^ UCL School of Pharmacy University College London London UK

**Keywords:** post‐colonoscopy, rectal cancer adenocarcinoma, antibiotics, colon cancer, interval cancer, rectal cancer

## Abstract

**Background:**

Antibiotics may alter colorectal cancer (CRC) risk due to gut dysbiosis. We aimed to study the specific and temporal effects of various antibiotics on CRC development in older individuals.

**Methods:**

This was a territory‐wide retrospective cohort study. Subjects aged 60 years and older who did not have CRC diagnosed on screening/diagnostic colonoscopy diagnosed between 2005 and 2013 were recruited. Exclusion criteria were history of CRC, colectomy, inflammatory bowel disease, and CRC diagnosed within 6 months of index colonoscopy. Exposure was use of any antibiotics up to 5 years before colonoscopy. The primary outcomes were CRC diagnosed >6 m after colonoscopy. Covariates were patient demographics, history of colonic polyps/polypectomy, concomitant medication use (NSAIDs, COX‐2 inhibitors, aspirin, and statins), and performance of endoscopy centers (colonoscopy volume and polypectomy rate). Stratified analysis was conducted according to nature of antibiotics and location of cancer.

**Results:**

Ninety seven thousand one hundred and sixty‐two eligible subjects (with 1026 [1.0%] cases of CRC) were identified, 58,704 (60.4%) of whom were exposed to antibiotics before index colonoscopy. Use of antibiotics was associated with a lower risk of cancer in rectum (adjusted hazard ratio [aHR]: 0.64, 95% CI: 0.54–0.76), but a higher risk of cancer in proximal colon (aHR: 1.63, 95%CI: 1.15–2.32). These effects differed as regards the anti‐anaerobic/anti‐aerobic activity, narrow‐/broad‐spectrum, and administration route of antibiotics.

**Conclusions:**

Antibiotics had divergent effects on CRC development in older subjects, which varied according to the location of cancer, antibiotic class, and administration route.

## Graphical abstract

Whether antibiotics modify colorectal cancer (CRC) risk remains controversial. In this study involving 97,162 patients aged ≥60 years who had index colonoscopy negative for CRC, antibiotic use was associated with 36% lower rectal cancer risk, 63% higher proximal colon cancer risk and neutral effect on distal colon risk. Our findings provide insight into the differential effects of various antibiotics on CRC development in older patients, which varied according to cancer locations, classes of antibiotics and routes of administration.

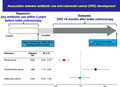

## 1 INTRODUCTION

Globally, colorectal cancer (CRC) is currently the 3rd most common cancer, and its incidence continues to rise in many developing regions including Asia.^1^ Although various factors including westernized diets have been attributed to rapid rise of colorectal cancer incidence, disruption of gut microbiota has been increasingly linked to the development of CRC.^2^ Antibiotics are among the potent modulators of intestinal microbiota. Several proposed mechanisms include an increase in carcinogenic microbes and loss of beneficial anaerobes^3^, ^4^ and weakening of host's immune defense against cancer.^5^ Intriguingly, antibiotics, in particular broad‐spectrum ones, affect outcomes of cancer patients receiving immunotherapy, possibly mediated by alteration of immune response related to the intestinal microbiota.^6^, ^7^

With continuous rise in global antibiotic consumption and an estimated annual consumption of 70 billion doses,^8^ it is important to address possible association between antibiotic exposure and CRC development. However, previous studies yielded inconsistent results. The Nurses' Health Study showed that antibiotics were associated with a higher risk of adenomas in proximal colon, but weak to no association for distal colon and rectum.^9^ While some epidemiological studies suggested increased CRC risk with prior antibiotic exposure,^10^, ^11^, ^12^ others did not identify such an association,^13^ and the risk was mainly limited to penicillins. Two recent studies showed that antibiotics were associated with reduced rectal cancer risk,^14^, ^15^ a finding which was not reported previously. However, this study was limited to oral form of antibiotics, and did not assess effects of different antibacterial spectrum on CRC risk. None of these studies investigated the role of antibiotics in sporadic CRC development in patients without CRC detected in previous colonoscopy.

Against this background, the aim of our study was to study effects of various antibiotics (including anti‐aerobic vs. anti‐anaerobic, narrow‐ vs. broad‐spectrum, and intravenous vs. oral) on development of sporadic CRC after baseline colonoscopy negative for CRC in a territory‐wide cohort of older subjects.

## 2 METHODS

### 2.1 Data source and study design

This was a retrospective cohort study based on data retrieved from clinical data analysis and reporting system (CDARS), a territory‐wide electronic database system possessed by the Hong Kong's Hospital Authority which is the sole public healthcare provider for about 90% of all healthcare services (including primary, secondary, and tertiary) of the local 7.5 million population. The CDARS contains patient demographics and clinical data including outpatient clinic visits, hospitalization, endoscopic and surgical procedures, investigation results, and drug dispensing history. Diagnoses are recorded by the International Classification of Diseases, Ninth Revision (ICD‐9) codes. Utilizing CDARS allows conduction of various large‐size cohort studies with >90% accuracy of coding.^16^, ^17^, ^18^, ^19^ We obtained ethics approval from the Institutional Review Board of the University of Hong Kong (HKU) and Hong Kong West Cluster (HKWC) of Hospital Authority. Inform consent was waived as patient confidentiality was protected by replacement of personal identity with reference key.

### 2.2 Study subjects and outcome of interest

eFigure Figure S1 illustrates the patient selection process. We identified subjects aged ≥60 years who had received colonoscopy between 2005 and 2013 in all public hospitals in our locality (i.e., index date). We specifically studied this older population because of their higher CRC risk and lower possibility of hereditary syndrome^20^ (the CDARS did capture data on this aspect). Exclusion criteria were history of CRC, colectomy, inflammatory bowel disease, and detected CRC (defined as CRC diagnosed within 6 months after index colonoscopy^21^).

The primary outcome of interest was all CRC that developed >6 m after index colonoscopy. Cancer subsites were grouped into proximal (cecum, ascending, and transverse colon [ICD‐9 codes: 153.4, 153.6, 153.0, 153.1]) and distal colon (splenic flexure, descending and sigmoid colon, and rectum [ICD‐9 codes: 153.2, 153.3, 153.7, 154.0, 154.1]). Our previous studies showed that coding accuracy of CRC from this database was 97.1%.^17^ Patients were followed from 6 months after colonoscopy and censored at diagnosis of CRC, death, or study end date (31 December 2017).

### 2.3 Exposure of interest and covariates

Exposure of interest was the use of any antibiotics before colonoscopy. Data of drug prescription and dispensing history were retrieved up to 5 years before colonoscopy. Antibiotic use was regarded as “ever use of any antibiotics” before the index colonoscopy as in the study by Zhang et al.^14^ These included 11 classes of antibiotics, namely penicillins, cephalosporins, quinolones, tetracyclines, carbapenems, macrolides, aminoglycosides, glycopeptides, nitroimidazoles, sulfa/trimethoprim, and other antibiotics (daptomycin, clindamycin, linezolid, nitrofurantoin, rifaximin, and rifampicin). Effects of nature of various antibiotics (anti‐aerobic vs anti‐anaerobic, narrow‐ vs. broad‐spectrum, and intravenous vs. oral) on CRC development were also studied. eTable S2 shows classification of antibiotics based on anti‐aerobic/anti‐anaerobic effect and antibacterial spectrum.

Other covariates taken into analysis were patient characteristics and performance of endoscopy centers (polypectomy rate and endoscopy volume annually).^21^, ^22^, ^23^, ^24^ Patient characteristics were age, sex, colonic polyp history, polypectomy at index colonoscopy, diseases related to alcoholism (gastrointestinal, hepatic, neurological, and psychiatric diseases), smoking (ICD‐9 code of V15.82 and chronic obstructive pulmonary disease [COPD]), comorbidities (neurological, cardiovascular, metabolic, hepatic, and renal diseases) (Table 1) and concomitant medication use (aspirin,^25^ non‐steroidal anti‐inflammatory drugs, cyclooxygenase [COX]‐2 inhibitors,^26^ and statins^27^, ^28^). eTable Figure S1 shows ICD‐9 codes of these diseases. Other medication use was defined ≥180‐day use as defined in previous study.^29^

**TABLE 1:** Characteristics of antibiotic and nonantibiotic users

	All (*n* = 97,162)	Antibiotic users (*n* = 58,704)	Nonantibiotic users (*n* = 38,458)
Age at index colonoscopy (years)^a^	71.4 (65.1–77.8)	72.7 (66.1–78.9)	69.5 (64.0–75.8)
Male sex (*n*, %)	50,841 (52.3%)	30,625 (52.2%)	20,216 (52.6%)
History of colonic polyp (*n*, %)	29,663 (30.5%)	19,666 (27.2%)	9997 (26.0%)
Polypectomy at index colonoscopy (*n*, %)	16,087 (16.6%)	10,027 (17.1%)	6060 (15.8%)
Smoking (*n*, %)	3637 (3.7%)	3332 (5.7%)	305 (0.8%)
Alcohol (*n*, %)	495 (0.5%)	389 (0.7%)	106 (0.3%)
DM (*n*, %)	14,032 (14.4%)	10,533 (17.9%)	3499 (9.1%)
Hypertension (*n*, %)	23,512 (24.2%)	17,468 (29.8%)	6044 (15.7%)
Dyslipidemia (*n*, %)	7290 (7.5%)	5112 (8.7%)	2178 (5.7%)
AF (*n*, %)	5029 (5.2%)	4098 (7.0%)	931 (2.4%)
IHD (*n*, %)	11,141 (11.5%)	8394 (14.3%)	2747 (7.1%)
CHF (*n*, %)	5737 (5.9%)	5045 (8.6%)	692 (1.8%)
Stroke (*n*, %)	6541 (6.7%)	5066 (8.6%)	1475 (3.8%)
CRF (*n*, %)	2986 (3.1%)	2664 (4.5%)	322 (0.8%)
Cirrhosis (*n*, %)	696 (0.7%)	593 (1.0%)	103 (0.3%)
Dementia (*n*, %)	1225 (1.3%)	1084 (1.8%)	141 (0.4%)
Parkinsonism (*n*, %)	726 (0.7%)	585 (1.0%)	141 (0.4%)
Aspirin (*n*, %)	22,004 (22.6%)	15,864 (27.0%)	6140 (16.0%)
NSAIDs (*n*, %)	8000 (8.2%)	5649 (9.6%)	2351 (6.1%)
COX‐2 inhibitors (*n*, %)	108 (0.1%)	71 (0.1%)	37 (0.1%)
Statins (*n*, %)	17,651 (18.2%)	11,629 (19.8%)	6022 (15.7%)
Annual center endoscopy volume^a^	2892 (2045–3316)	2892 (2054–3316)	2887 (2033–3291)
Annual center polypectomy rate^a^	24.6% (21.7%–28.2%)	24.6% (21.7%–28.0%)	24.7% (21.6%–28.4%)

Abbreviations: AF, atrial fibrillation; CHF, congestive heart failure; COX‐2, cyclooxygenase‐2; CRF, chronic renal failure; DM, diabetes mellitus; IHD, ischemic heart disease; NSAIDs, non‐steroidal anti‐inflammatory drugs.

^a^ Expressed as median (years) with interquartile range.

To investigate duration‐response relationship, antibiotic use duration was categorized into three groups (1) never use, (2) < median, and (3) ≥ median.

### 2.4 Statistical analysis

We conducted statistical analysis by using R version 3.2.3 (R Foundation for Statistical Computing) statistical software. Continuous variables were expressed as median and interquartile range (IQR). Mann–Whitney *U* test was applied to compare difference in continuous variables between two groups. Chi‐square test was used for categorical variables. Multivariable Cox proportional hazards model was applied to calculate the adjusted hazard ratio (aHR) of CRC with antibiotic use. Stratified analysis was conducted as regards the location of cancer (proximal and distal colon and rectum). Association between CRC and various antibiotics as well as antibiotic nature (anti‐aerobic vs. anti‐anaerobic, narrow‐ vs. broad‐spectrum, and intravenous vs. oral) on CRC was also determined. Subgroup analysis was conducted as regards sex, diabetes mellitus (DM), and history of colonic polyps/polypectomy. Statistical significance is defined with a two‐sided *p*‐value of <0.05.

## 3 RESULTS

### 3.1 Characteristics of study cohort

Ninety seven thousand one hundred and sixty‐two (male: 50,841 [52.3%]) subjects fulfilled the eligibility criteria (eFigure Figure S1), totaling 797,107 person‐years. They underwent index colonoscopy at a median age of 71.4 years (IQR: 65.1–77.8) (Table 1).

One thousand twenty‐six (1.1%) subjects were diagnosed to have CRC after colonoscopy (rectum: 601 [58.6%], proximal colon: 171 [16.7%], and distal colon: 254 [24.8%]), at an incidence rate of 2.4 per 10,000 person‐years. They were diagnosed with CRC at a median age of 79.1 years (IQR: 72.9–85.0), with interval between index colonoscopy and cancer diagnosis being a median of 1.8 years (IQR:1.0–3.6).

### 3.2 Association between pre‐colonoscopy antibiotic use and CRC

Antibiotic users had use of antibiotics for a median of 15 days (IQR:7–31). Antibiotic use before index colonoscopy was associated with a lower rectal cancer risk (aHR:0.64, 95% CI:0.54–0.76) but higher cancer risk in proximal colon (aHR:1.63, 95% CI:1.15–2.32) (Table 2). Antibiotics had no effect on the risk of cancer development in distal (aHR:0.99, 95% CI:0.76–1.30). Compared with antibiotic non‐use, aHR of cancer in rectum was 0.73 (95% CI:0.59–0.91) and 0.58 (95% CI:0.48–0.71) with antibiotic use <2 weeks and ≥2 weeks, respectively (Table 3). On the contrary, aHRs of cancer in proximal colon was 1.73 (95% CI:1.15–2.60) and 1.57 (95% CI:1.07–2.30) for antibiotic use <2 weeks and ≥2 weeks, respectively.

**TABLE 2:** Association between colorectal cancer development and antibiotics after index colonoscopy negative for colorectal cancer

	Number of subjects and colorectal cancer cases	Adjusted hazard ratio^a^	95% confidence interval	*p*‐value
Rectum (number = 96,737, cancer cases = 601)				
Antibiotic non‐use	Number = 38,372; Cancer = 287	Reference	–	–
Any antibiotic use	Number = 58,480; Cancer = 314	0.64	0.54–0.76	<0.001
Proximal colon (Number = 96,307, Cancer cases = 171)				
Antibiotic non‐use	Number = 38,080; Cancer = 45	Reference	–	–
Any antibiotic use	Number = 58,227; Cancer = 126	1.63	1.15–2.32	0.006
Distal colon (Number = 96,390, Cancer cases = 254)				
Antibiotic non‐use	Number = 38,126; Cancer = 91	Reference	–	–
Any antibiotic use	Number = 58,264; Cancer = 163	0.99	0.76–1.30	0.965

^a^ Adjusted for age, sex, colonic polyp history, polypectomy at index colonoscopy, alcohol‐related diseases, smoking, other comorbidities (hypertension, dyslipidemia, diabetes mellitus, ischemic heart disease, congestive heart failure, atrial fibrillation, stroke, cirrhosis, chronic renal failure, parkinsonism, and dementia) and concurrent medications (aspirin, COX‐2 inhibitors, and statins), annual center polypectomy rate, and endoscopy volume.

**TABLE 3:** Association between colorectal cancer and duration of antibiotic use

	Number of subjects and colorectal cancer cases	Adjusted hazard ratio^a^	95% confidence interval	*p*‐value
Rectum (number = 96,737, cancer cases = 601)				
Antibiotic non‐use	Number = 38,372; Cancer = 287	Reference	–	–
<2 weeks	Number = 21,587; Cancer = 127	0.73	0.59–0.91	0.004
≥2 weeks	Number = 36,828; Cancer = 187	0.58	0.48–0.71	<0.001
Proximal colon (number = 96,307, cancer cases = 171)				
Antibiotic non‐use	Number = 38,080; cancer = 45	Reference	–	–
<2 weeks	Number = 21,509; cancer = 49	1.73	1.15–2.60	0.008
≥2 weeks	Number = 36,718; cancer = 77	1.57	1.07–2.30	0.021
Distal colon (number = 96,390, cancer cases = 254)				
Antibiotic non‐use	Number = 38,126; cancer = 91	Reference	–	–
<2 weeks	Number = 21,512; cancer = 52	0.92	0.65–1.30	0.634
≥2 weeks	Number = 36,752; cancer = 111	1.04	0.78–1.40	0.786

^a^ Adjusted for age, sex, colonic polyp history, polypectomy at index colonoscopy, alcohol‐related diseases, smoking, other comorbidities (hypertension, dyslipidemia, diabetes mellitus, ischemic heart disease, congestive heart failure, atrial fibrillation, stroke, cirrhosis, chronic renal failure, parkinsonism, and dementia) and concurrent medications (aspirin, COX‐2 inhibitors, and statins), annual center polypectomy rate, and endoscopy volume.

### 3.3 Association between CRC and different classes of antibiotics

When compared to non‐antibiotic use, penicillins were associated with a lower risk of CRC (aHR:0.83, 95% CI:0.72–0.95) and aminoglycosides were associated with a higher risk (aHR:1.53, 95% CI:1.05–2.25), while there was no significant association between CRC and other antibiotic (Table 4). Further analysis showed that the beneficial effect of penicillins was limited to rectum (aHR: 0.68, 95% CI:0.87–0.81) and the harmful effect of aminoglycosides was limited to distal colon (aHR: 2.13, 95% CI:1.21–3.77) (eTable S3).

**TABLE 4:** Association between colorectal cancer development and different antibiotic classes

	Adjusted hazard ratio^a^	95% confidence interval	*p*‐value
Penicillins	0.83	0.73–0.96	0.009
Cephalosporins	0.97	0.81–1.16	0.739
Macrolides	1.06	0.87–1.28	0.579
Carbapenems	1.16	0.69–1.95	0.574
Quinolones	0.91	0.75–1.09	0.297
Tetracyclines	0.52	0.25–1.10	0.088
Aminoglycosides	1.53	1.05–2.25	0.029
Nitromidazoles	1.03	0.83–1.27	0.818
Glycopeptides	0.64	0.31–1.31	0.220
Sulfa and trimethoprim	0.70	0.43–1.12	0.548
Others (clindamycin, nitrofurantoin, linezolid, rifampicin, rifaximin, daptomycin)	0.92	0.70–1.20	0.132

^a^ Adjusted for age, sex, colonic polyp history, polypectomy at index colonoscopy, alcohol‐related diseases, smoking, other comorbidities (hypertension, dyslipidemia, diabetes mellitus, ischemic heart disease, congestive heart failure, atrial fibrillation, stroke, cirrhosis, chronic renal failure, parkinsonism, and dementia), and concurrent medications (aspirin, COX‐2 inhibitors, and statins), annual center polypectomy rate, and endoscopy volume.

### 3.4 Antibiotic nature on the risk of CRC development

Analysis was limited to rectal and proximal cancer since antibiotics did not affect distal colon cancer risk. Table 5 shows the effects of different antibiotics on CRC according to (1) anti‐aerobic versus anti‐anaerobic activity, (2) narrow‐ versus broad‐spectrum activity, and (3) intravenous versus oral versus both administration routes.

**TABLE 5:** Association between nature of antibiotics and CRC as regards anatomical location

	Number of subjects and colorectal cancer cases	Adjusted hazard ratio^a^	95% confidence interval	*p*‐value
Rectum (Number = 96,737, Cancer = 601)				
Anti‐aerobic vs anti‐anaerobic activity				
Antibiotic non‐use	Number = 38,322; Cancer = 287	Reference	**–**	**–**
Anti‐aerobic	Number = 8034; Cancer = 43	0.68	0.49–0.93	0.017
Anti‐anaerobic	Number = 50,381; Cancer = 271	0.64	0.53–0.76	<0.001
Narrow‐ vs broad‐spectrum activity				
Antibiotic non‐use	Number = 38,322; Cancer = 287	Reference	–	–
Narrow‐spectrum	Number = 3543; Cancer = 20	0.77	0.49–1.22	0.277
Broad‐spectrum	Number = 54,872; Cancer = 294	0.63	0.53–0.75	< 0.001
Intravenous vs. oral antibiotics				
Antibiotic non‐use	Number = 38,322; Cancer = 287	Reference	–	–
Intravenous	Number = 3481; Cancer = 23	0.77	0.50–1.18	0.237
Oral	Number = 34,690; Cancer = 178	0.65	0.54–0.78	< 0.001
Both (intravenous and oral)	Number = 20,244; Cancer = 113	0.60	0.47–0.76	< 0.001
Proximal colon (*n* = 96,307, Cancer = 171)				
Anti‐aerobic vs. anti‐anaerobic activity				
Antibiotic non‐use	Number = 38,080; Cancer = 45	Reference	**–**	**–**
Anti‐aerobic	Number = 8005; Cancer = 14	1.33	0.73–2.43	0.354
Anti‐anaerobic	Number = 50,222; Cancer = 112	1.69	1.18–2.41	0.004
Narrow‐ vs. broad‐spectrum activity				
Antibiotic non‐use	Number = 38,080; Cancer = 45	Reference	–	–
Narrow‐spectrum	Number = 3532; Cancer = 9	2.08	1.02–4.27	0.045
Broad‐spectrum	Number = 54,695; Cancer = 117	1.60	1.13–2.29	0.009
Intravenous vs oral antibiotics				
Antibiotic non‐use	Number = 38,080; Cancer = 45	Reference	–	–
Intravenous	Number = 3470; Cancer = 12	2.61	1.37–4.97	0.004
Oral	Number = 34,580; Cancer = 68	1.53	1.05–2.24	0.028
Both (intravenous and oral)	Number = 20,177; Cancer = 46	1.65	1.07–2.56	0.024

^a^ Adjusted for age at which index colonoscopy was performed, sex, history of colonic polyps, polypectomy at index colonoscopy, alcohol consumption, smoking, other comorbidities (hypertension, dyslipidemia, diabetes mellitus, ischemic heart disease, congestive heart failure, atrial fibrillation, stroke, cirrhosis, chronic renal failure, parkinsonism, and dementia) and concurrent medications (aspirin, COX‐2 inhibitors, and statins), annual center endoscopy volume, and center polypectomy rate.

#### 3.4.1 Rectal cancer

Both anti‐aerobic and anti‐anaerobic antibiotics were associated with lower risk of cancer in rectum (aHR:0.68, 95% CI:0.49–0.93 and aHR:0.64, 95% CI:0.53–0.76, respectively). While narrow‐spectrum antibiotics had no effect on rectal cancer.

(aHR:0.77, 95% CI:0.49–1.22), broad‐spectrum antibiotics were associated with a lower risk of cancer in rectum (aHR:0.63, 95% CI:0.53–0.75). Different routes of administration yielded similar results (intravenous:0.77 [95% CI:0.50–1.18], oral:aHR 0.65 [95% CI:0.54–0.78], and both routes:0.60 [95% CI:0.47–0.76]).

#### 3.4.2 Proximal colon cancer

While anti‐aerobic antibiotics did not increase the risk of cancer in proximal colon (aHR:1.33, 95% CI:0.73–2.43), anti‐anaerobic antibiotics were associated with increased risk (aHR:1.69, 95% CI:1.18–2.41). Both narrow‐ and broad‐spectrum antibiotics were associated with raised risk of proximal colon cancer (aHR:2.08, 95% CI:1.02–4.27; and aHR:1.60, 95% CI:1.13–2.29, respectively). The aHR of intravenous and oral antibiotics was 2.61 (95% CI:1.37–4.97) and 1.53 (95% CI:1.05–2.24), respectively.

### 3.5 Subgroup analysis of antibiotics on CRC

eTable S4 shows that the protective effects of antibiotics on rectal cancer persisted in different subgroups with similar effect magnitudes even after stratification according to patient's sex, history of DM, and colonic polyps. For proximal colon cancer, the harmful effects of antibiotics were limited to males (aHR:2.28, 95% CI:1.34–3.88), nondiabetic patients (aHR:1.67, 95% CI: 1.17–2.43), and patients with colonic polyp history (aHR:1.85, 95% CI: 1.12–3.04).

## 4 DISCUSSION

This is the first comprehensive study, which involved >90,000 older subjects, to demonstrate the differential effects of pre‐colonoscopy antibiotics use on subsequent sporadic CRC risk according to cancer location after negative baseline colonoscopy. We found that antibiotics had divergent effects on cancer development in rectum (36% lower risk) and proximal colon (63% higher risk), but relatively neutral effect in distal colon. The associations also varied with different classes or spectrum of antibiotics, with penicillins and aminoglycosides being associated with lower and higher CRC risk, respectively. Further analysis showed that both anti‐anaerobic and anti‐aerobic antibiotics were associated with lower rectal cancer risk, but only anti‐anaerobic antibiotics were associated with higher proximal cancer risk.

Antibiotics exert differential effects on proximal and distal cancer development was only recently reported in an epidemiological study from the United Kingdom.^14^ In their case–control study, antibiotics were associated with higher proximal colon cancer risk, particularly with anti‐anaerobic antibiotics, but lower risk in rectum. As yet, our study was different from Zhang's study by including older patients only who were more likely to have sporadic CRC. Moreover, our cohort design included all patients who had baseline colonoscopies that were negative for cancer rather than case–control design. Third, we accounted for antibiotics that were given by intravenous route and not limited to oral antibiotics only. Lastly, CRCs were predominantly rectal cancer (58.6%) in our study when compared to the UK study (rectal cancer 31.9%). Despite the difference in study design, patient's ethnic origin, and even cancer characteristics, both studies demonstrated similar divergent effects of antibiotics regarding the risks of cancer in proximal colon and rectum.

The reasons behind the differential effects of antibiotics on CRC remains perplexing. Gut dysbiosis induced by antibiotics has been linked with CRC development due to a possible increase in pro‐neoplastic microbiota and loss of health‐promoting anaerobes.^3^, ^4^ For instance, increase in abundance of colibactin‐producing *Escherichia coli*, *Fusobacterium nucleatum*, and enterotoxigenic *Bacteroides fragilis* in CRC, with depletion of butyrate‐producing microbes are reported in CRC.^30^, ^31^ Animal models also showed that antibiotics could affect the metabolome‐related carcinogenesis pathway,^32^ including reduction in short‐chain fatty acids levels from microbial fermentation which would disrupt cell proliferation/apoptosis, regulation of inflammation, and immune response. Bacterial translocation from antibiotic also could increase intestinal permeability and activate immune system with ensuing chronic inflammation.^33^ However, it remains unknown to show effects of antibiotics on CRC development could be as topical as shown in the current study. The proximal colon is the first site of exposure to incompletely absorbed antibiotics, and the impact of antibiotics on polymicrobial invasive biofilms may therefore be more prominent in the proximal colon.^34^, ^35^ The different distribution of various microbes along the colorectun continuum is another possible reason accounting for the divergent outcomes. A previous study showed that the density gradient of *F. nucleatum* decreased from proximal colon to rectum.^36^ It has also been shown that the bacterial activity of *Bacteroidetes, Firmicutes,* and *Actinobacteria* (which produce health‐promoting products like acetate, propionate, and butyrate) are the highest in proximal colon, and fermentation of bacterial proteins and amino acids into toxic metabolites (e.g., branched‐chain fatty acids) take place in distal colon.^37^ It has been postulated that host factors involved in immune and microbial homeostasis reduce caudally, hence favoring proliferation of pro‐neoplastic microbes to increase the susceptibility of the distal colorectum to injury. This notion is illustrated by a recent study that dietary pattern that promote sulfur‐metabolizing microbes increased cancer risk in distal colorectum but not proximal colon.^38^ In addition, difference in embryonic origin of distal and proximal colon could partly explain different molecular and clinicopathological characteristics of CRCs, and hence antibiotic effects.^39^

In this study, longer duration (>2 weeks) of antibiotic use associated with even lower risk of rectal cancer, and similar duration effects of antibiotics were not observed in proximal cancer. It remains uncertain whether proximal colon is more susceptible to antibiotics than the rectum, and hence even 2 weeks may be sufficient to cause disruption of proximal colon microbiota. This is consistent with the study findings by Zhang et al^14^ which reported that proximal colon cancer risk increased after minimal antibiotic use (less than 2 weeks) and reached a plateau after 60 days of cumulative exposure (p‐non‐linear = 0.0179). Although gut microbiota and metabolites may be affected even by short‐term antibiotic usage, false‐negative colonoscopy (in particular proximal colon), and ascertainment/detection bias may bias the result. False‐negative colonoscopy was a concern as the majority of our patients develop CRC within 4 years after negative baseline colonoscopy. Ascertainment/detection bias in which antibiotic users were less healthy with more frequent colonoscopy may result in earlier cancer diagnosis.

In our study, different effects on CRC were observed in antibiotics with different antibacterial spectrum and route of administration. Notably, while both anti‐aerobic and anti‐anaerobic antibiotics were associated with lower rectal cancer, there was statistical significance for broad‐spectrum and oral antibiotics only. In contrast, while there was a higher risk of proximal colon cancer regardless of antibiotic spectrum or route, statistical significance was present for anti‐anaerobic antibiotics only. Nevertheless, numerically the direction of effect appears to be consistent as regards the antibiotics nature and route, and underpower was a possibility due to subgroup analysis.

Our subgroup analysis also shows that protective effects of antibiotics on rectal cancer did not differ with patient's sex, presence of DM, or history of colonic polyps. On the other hand, harmful effects of antibiotics in proximal colon cancer were limited to males, nondiabetic patients, and subjects with colonic polyp history.

The inhibition of colibactin‐producing bacteria^30^, ^31^ may explain a protective effect of penicillins, in particular proximal colon. Aminoglycosides were found to be associated with higher risk of colon cancer, in particular distal colon, which may be related to lower levels of short‐chain fatty acids from microbial fermentation.^32^ Further studies are necessary to identify the underlying mechanisms of these discrepancies.

A number of strengths existed for this study. First, our territory‐wide database enables the comprehensive capture of diagnoses and drug history, minimizing biases inherent to observational studies such as recall and selection biases.^25^ Reverse causality was largely avoided as there was a minimum of 6‐month lapse between last antibiotic use and CRC development due to exclusion of cancer ≤6 m after index colonoscopy. Immortal time bias also did not exist as antibiotic exposure was defined before index colonoscopy.

Our study had a few limitations. First, some data of CRC risk factors such as lifestyle factors and family history were not available. Nevertheless, given the similar proportion of patients with history of colonic polyps and polypectomy between antibiotic users and non‐users, a significant difference was unlikely. Moreover, we included older subjects who were less likely to suffer from hereditary cancer. Use of diagnosis codes to identify smoking and alcoholism could also underestimate their true prevalence. Second, quality metrics of index colonoscopy (e.g., quality of bowel preparation, adenoma detection rate of endoscopists) were not recorded in the database. To overcome this, we used polypectomy rates and colonoscopy volume of endoscopy centers as surrogate markers of center's performances for adjustment in multivariable analysis. Third, mechanisms and reasons of the CRC could not be ascertained, precluding further investigation into how antibiotics modify early CRC development after index colonoscopy. Notably, false‐negative colonoscopy and ascertainment/detection bias may affect the study results. However, divergent effects of antibiotics as regards different colonic segments and nature of antibiotics makes this concern less substantiated. In addition, adjustment for an extensive set of comorbidities minimized this possible bias.

## 5 CONCLUSION

In this study of >90,000 subjects with baseline colonoscopy negative for CRC, a lower risk of cancer in rectum but higher risk in proximal colon was observed with antibiotic use. This observation differed as regards the class and spectrum of antibiotics. Future studies should focus on the interaction between antibiotics and gut microbiota on the development of CRC.

## CONFLICT OF INTEREST

EWC has received funding from Bayer, Bristol‐Myers Squibb, Pfizer, and Takeda, for work unrelated to this study. WKS has received speaker fees from AbbVie, Eisai, Mylan, and Gilead Sciences, honorarium for attending advisory board for AbbVie, Celltrion, CSL Behring, and Gilead Sciences, and received research funding from Echosens and Gilead Sciences but not related to the current study.

ICKW has received grants from Janssen, Pfizer, Bayer, Amgen, and Novartis but not related with the present study. WKL has received speaker fee from Eisai, Ipsen, and honorarium for attending advisory board for Janssen and Pfizer.

## AUTHOR CONTRIBUTION

Dr. Ka Shing Cheung was involved with study concept and design; analysis and interpretation of data; drafting of manuscript; and approval of the final version of the manuscript. Dr. Esther W Chan, Ms Lijia Chen, Dr. Anthony Tam, and Dr. Wai Kay Seto were involved with acquisition of data; critical revision of the manuscript for important intellectual content; and approval of the final version of the manuscript. Dr. Irene OL Wong was involved in critical revision of the manuscript for important intellectual content; Professors Ian CK Wong and Wai K Leung were involved with the study concept and design; analysis and interpretation of data; drafting of manuscript; critical revision of the manuscript for important intellectual content; study supervision; and approval of the final version of the manuscript.

## ETHICS STATEMENT

We obtained ethics approval from the Institutional Review Board of the University of Hong Kong (HKU) and Hong Kong West Cluster (HKWC) of Hospital Authority. Inform consent was waived as patient confidentiality was protected by replacement of personal identity with reference key.

## Supporting information


Figure S1

## Data Availability

Our study retrieved data from electronic healthcare database and id not include patients as study participants. We will disseminate the results via general media, but not directly to patients.
