# Supplementary material for: Association between antibiotic consumption and colon and rectal cancer development in older individuals: A territory‐wide study
Source: Cancer Med. 2022 Apr 29;11(20):3863–72. doi: 10.1002/cam4.4759 (PMC9582694; doi:10.1002/cam4.4759)
Supplement: Supplementary file 1 — Figure S1 [file CAM4-11-3863-s001.pdf]

eFigure 1. Patient selection flow diagram

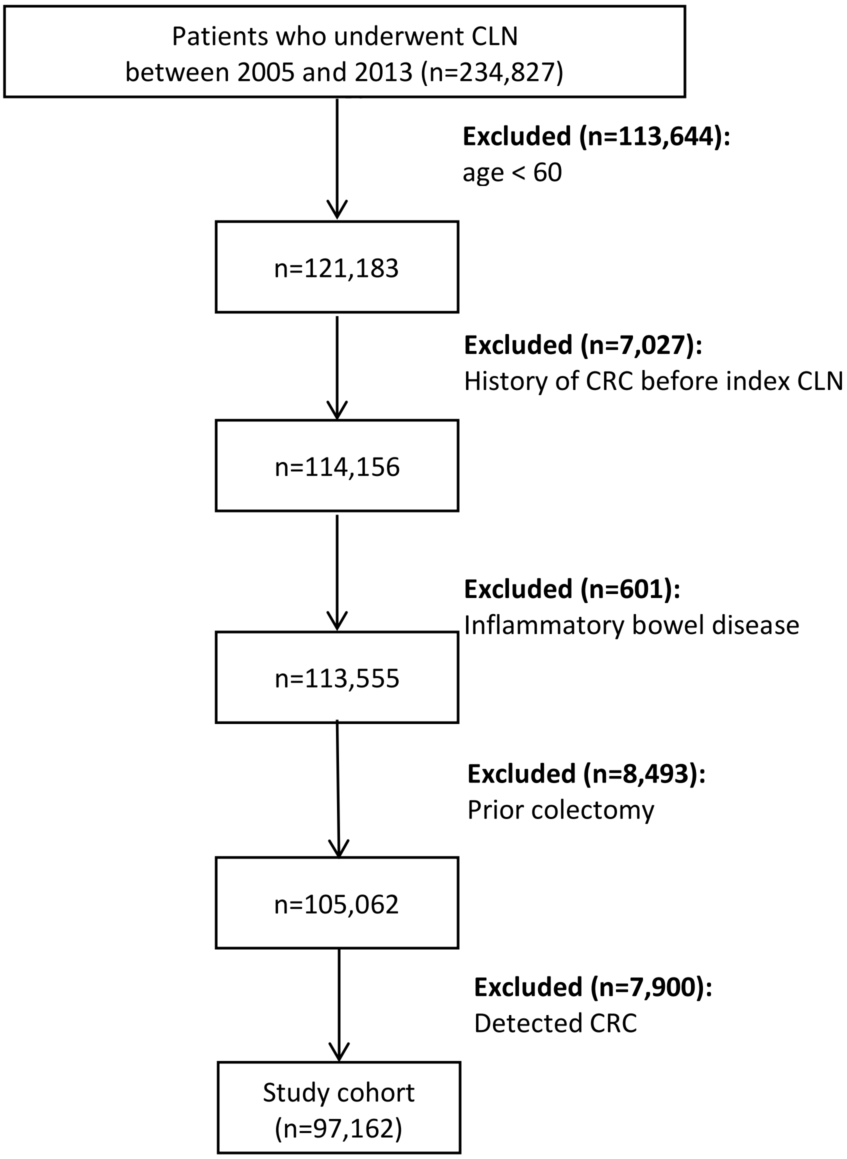

eTable 1. ICD-9 codes for covariates

**Covariates**	
**Lifestyle factors**	
Smoking*	491, 492, 496, V15.82
Alcohol*	291, 303, 305.0, 571.0, 571.1, 571.2, 571.3, 980.8, 980.9
**Cardiovascular and metabolic risk factors**	
Obesity	278.0, 278.1
Diabetes mellitus	249, 250
Hypertension	401-405
Dyslipidemia	272.0-272.4
**Cardiovascular diseases**	
Ischemic heart disease	410-413, 414.0, 414.8, 414.9, 429.7
Atrial fibrillation	427.3
Congestive heart failure	402.01, 402.11, 402.91, 404.01, 404.03, 404.11, 404.13, 404.91, 404.93, 428
Stroke	430-432, 433.01, 433.11, 433.21, 433.31, 433.81, 433.91, 434.01, 434.11, 434.91, 436, 437.0, 437.1
**Renal and liver diseases**	
Chronic renal failure	585
Cirrhosis	571.2, 571.5, 571.6, 572.2-572.4, 573.5
**Neurological diseases**	
Parkinsonism	332
Dementia	290, 291.2, 292.82, 294.1-294.2
**Gastrointestinal diseases**	
Inflammatory bowel disease	555, 556
Colectomy	45.8, 45.81, 45.82, 45.83, V45.89
* Smoking was identified by the ICD-9 code of V15.82 and by the proxy of chronic obstructive pulmonary disease. Heavy alcohol consumption was inferred from the presence of alcohol-related disorders, including hepatic, gastrointestinal, neurological and psychiatric diseases.	

eTable 2. Anti-anaerobic/anti-aerobic activity and anti-bacterial spectrum of various classes of antibiotics

**Class**	**Route**	**Anti-anaerobic/anti-aerobic activity**	**Anti-bacterial spectrum**
Penicillins			
Benzylpenicillin	Intravenous	Anti-aerobic	Narrow spectrum
Phenoxymethylpenicillin	Oral	Anti-aerobic	Narrow spectrum
Benzathine penicillin G	Intramuscular	Anti-aerobic	Narrow spectrum
Penicillin G procaine	Intramuscular	Anti-aerobic	Narrow spectrum
Cloxacillin	Oral	Anti-aerobic	Narrow spectrum
	Intravenous	Anti-aerobic	Narrow spectrum
Flucloxacillin	Oral	Anti-aerobic	Narrow spectrum
	Intravenous	Anti-aerobic	Narrow spectrum
Amoxycillin +/- clavulanate	Oral	Anti-anaerobic	Broad spectrum
	Intravenous	Anti-anaerobic	Broad spectrum
Ampicillin +/- sulbactam	Oral	Anti-anaerobic	Broad spectrum
	Intravenous	Anti-anaerobic	Broad spectrum
Piperacillin +/- tazobactam	Oral	Anti-anaerobic	Broad spectrum
	Intravenous	Anti-anaerobic	Broad spectrum
Ticarcillin + clavulanate	Oral	Anti-anaerobic	Broad spectrum
	Intravenous	Anti-anaerobic	Broad spectrum
Cephaloporins			
Cefazolin	Intravenous	Anti-aerobic	Narrow spectrum
Cefuroxime	Oral	Anti-aerobic	Broad spectrum
	Intravenous	Anti-aerobic	Broad spectrum
Ceftibuten	Oral	Anti-aerobic	Broad spectrum
Ceftazidime	Intravenous	Anti-aerobic	Broad spectrum
Ceftriaxone	Intravenous	Anti-aerobic	Broad spectrum
	Intramuscular	Anti-aerobic	Broad spectrum
Cefotaxime	Intravenous	Anti-aerobic	Broad spectrum
Cefepime	Intravenous	Anti-aerobic	Broad spectrum
Cefaclor	Oral	Anti-anaerobic	Broad spectrum
Cefoperazone + Sulbactam	Intravenous	Anti-anaerobic	Broad spectrum
Cefoxitin	Intravenous	Anti-anaerobic	Broad spectrum
Ceftaroline	Intravenous	Anti-anaerobic	Broad spectrum
Macrolides			
Azithromycin	Oral	Anti-aerobic	Narrow spectrum
	Intravenous	Anti-aerobic	Narrow spectrum
Erythromycin	Oral	Anti-aerobic	Narrow spectrum
	Intravenous	Anti-aerobic	Narrow spectrum
Clarithromycin	Oral	Anti-aerobic	Narrow spectrum
	Intravenous	Anti-aerobic	Narrow spectrum
Carbapenems			
Imipenem + cilastatin sodium	Intravenous	Anti-anaerobic	Broad spectrum
Ertapenem	Intravenous	Anti-anaerobic	Broad spectrum
Meropenem	Intravenous	Anti-anaerobic	Broad spectrum

Monobactams			
Aztreonam	Intravenous	Anti-aerobic	Narrow spectrum
Quinolones			
Ciprofloxacin	Oral	Anti-aerobic	Broad spectrum
	Intravenous	Anti-aerobic	Broad spectrum
Levofloxacin	Oral	Anti-aerobic	Broad spectrum
	Intravenous	Anti-aerobic	Broad spectrum
Moxifloxacin	Oral	Anti-anaerobic	Broad spectrum
	Intravenous	Anti-anaerobic	Broad spectrum
Tetracyclines			
Tetracycline	Oral	Anti-aerobic	Broad spectrum
	Intravenous	Anti-aerobic	Broad spectrum
Doxycycline	Oral	Anti-aerobic	Broad spectrum
	Intravenous	Anti-aerobic	Broad spectrum
Minocycline	Oral	Anti-aerobic	Broad spectrum
	Intravenous	Anti-aerobic	Broad spectrum
Tigecyclines	Intravenous	Anti-anaerobic	Broad spectrum
Oxytetracycline	Oral	Anti-aerobic	Broad spectrum
	Intravenous	Anti-aerobic	Broad spectrum
	Intramuscular	Anti-aerobic	Broad spectrum
Aminoglycosides			
Gentamicin	Intravenous	Anti-aerobic	Broad spectrum
Amikacin	Intravenous	Anti-aerobic	Broad spectrum
Neomycin	Oral	Anti-aerobic	Broad spectrum
Tobramycin	Oral	Anti-aerobic	Broad spectrum
	Intravenous	Anti-aerobic	Broad spectrum
Streptomycin	Intravenous	Anti-aerobic	Narrow spectrum
Nitroimidazoles			
Metronidazole	Oral	Anti-anaerobic	Broad spectrum
	Intravenous	Anti-anaerobic	Broad spectrum
Tinidazole	Oral	Anti-anaerobic	Broad spectrum
Glycopeptides			
Vancomycin	Oral	Anti-aerobic	Narrow spectrum
	Intravenous	Anti-aerobic	Narrow spectrum
Teicoplanin	Intravenous	Anti-aerobic	Narrow spectrum
Others			
**Clindamycin**	Oral	Anti-anaerobic	Narrow spectrum
	Intravenous	Anti-anaerobic	Narrow spectrum
Linezolid	Oral	Anti-anaerobic	Narrow spectrum
	Intravenous	Anti-anaerobic	Narrow spectrum
Septrin	Oral	Anti-anaerobic	Broad spectrum
	Intravenous	Anti-anaerobic	Broad spectrum
Nitrofurantoin	Oral	Anti-aerobic	Narrow spectrum
Rifampicin	Oral	Anti-anaerobic	Broad spectrum
	Intravenous	Anti-anaerobic	Broad spectrum
Rifaximin	Oral	Anti-anaerobic	Broad spectrum
Daptomycin	Intravenous	Anti-aerobic	Narrow spectrum

eTable 3. Subgroup analysis of the association between penicillins, aminoglycosides on CRC risk according to cancer subsite

	Adjusted hazard ratio*	95% confidence interval	p-value
**Penicillins**			
Rectum	0.68	0.87 – 0.81	< 0.001
Distal colon	0.96	0.74 – 1.24	0.738
Proximal colon	1.34	0.97 – 1.83	0.072
**Aminoglycosides**	0.91	0.75 – 1.09	0.297
Rectum	1.07	0.64 – 1.80	0.796
Distal colon	2.13	1.21 – 3.77	0.009
Proximal colon	0.82	0.26 – 2.57	0.729

* Adjusted for age, sex, colonic polyp history, polypectomy at index colonoscopy, alcohol-related diseases, smoking, other comorbidities (hypertension, dyslipidemia, diabetes mellitus, ischemic heart disease, congestive heart failure, atrial fibrillation, stroke, cirrhosis, chronic renal failure, parkinsonism, dementia) and concurrent medications (aspirin, COX-2 inhibitors, statins), annual center polypectomy rate and endoscopy volume

eTable 4. Subgroup analysis of the association between antibiotics and CRC according to cancer subsite

	Number of patients and events	aHR	95% CI	P-value
**Rectum (n=96,737, event=601)**				
Sex				
Male	n=50,597; event=350	0.60	0.48 – 0.75	< 0.001
Female	n=46,140; event=251	0.70	0.54 – 0.91	0.008
Diabetes mellitus				
Yes	n=13,979; event=70	0.60	0.36 – 1.02	0.057
No	n=82,758; event=531	0.65	0.54 – 0.77	< 0.001
History of colonic polyps or polypectomy				
Yes	n=28,979; event=176	0.69	0.51 – 0.95	0.024
No	n=67,758; event=425	0.62	0.51 – 0.76	< 0.001
**Proximal colon (n=96,307, event=171)**				
Sex				
Male	n=50,333; event=86	2.28	1.34 – 3.88	0.002
Female	n=45,974; event=85	1.19	0.74 – 1.91	0.468
Diabetes mellitus				
Yes	n=13,929; event=20	1.17	0.38 – 3.58	0.788
No	n=82,378; event=151	1.69	1.17 – 2.43	0.005
History of colonic polyps or polypectomy				
Yes	n=28,898; event=95	1.85	1.12 – 3.04	0.016
No	n=67,409; event=76	1.45	0.88 – 2.39	0.142
**Distal colon (n=96,390, event=254)**				
Sex				
Male	n=50,405; event=158	1.12	0.79 – 1.59	0.520
Female	n=45,985; event=96	0.81	0.53 – 1.24	0.343
Diabetes mellitus				
Yes	n=13,942; event=33	1.74	0.66 – 4.61	0.262
No	n=82,448; event=221	0.94	0.70 – 1.25	0.659
History of colonic polyps or polypectomy				
Yes	n=28,939; event=135	0.94	0.65 – 1.37	0.758
No	n=67,452; event=119	1.05	0.71 – 1.55	0.805

* Adjusted for age at which index colonoscopy was performed, sex, history of colonic polyps, polypectomy at index colonoscopy, smoking status, alcohol consumption, other comorbidities (diabetes mellitus, hypertension, dyslipidemia, atrial fibrillation, ischemic heart disease, congestive heart failure, stroke, chronic renal failure, cirrhosis, dementia, parkinsonism) and concurrent medications (aspirin, cyclooxygenase-2 inhibitors, statins), annual center endoscopy volume and center polypectomy rate

Abbreviations: CRC, colorectal cancer; aHR, adjusted hazard ratio; 95% CI, 95% confidence interval
